# Characterisation of a novel cold‐adapted calcium‐activated transglutaminase: implications for medicine and food processing

**DOI:** 10.1002/2211-5463.12826

**Published:** 2020-03-16

**Authors:** Rebeca Garcia Alvarez, Pralav Karki, Ida Elise Langleite, Ragna‐Johanne Bakksjø, Lutz Andreas Eichacker, Clemens Furnes

**Affiliations:** ^1^ Centre for Organelle Research Faculty of Science and Technology University of Stavanger Stavanger Norway

**Keywords:** Atlantic cod, calcium‐dependent, cold adaption, recombinant expression, transglutaminase

## Abstract

Transglutaminases are a family of enzymes that catalyse the cross‐linking of proteins by forming covalent bonds between lysine and glutamine residues in various polypeptides. Cross‐linking reactions are involved in blood clots, skin formation, embryogenesis and apoptosis. Clinically, these enzymes appear to be implicated in neurodegenerative diseases, tumours and coeliac diseases. Transglutaminases have great potential for use in the food industry because of their ability to cross‐link proteins that are not normally linked. Here, a gene coding for transglutaminase from Atlantic cod was cloned into a bacterial expression vector and used to transform protein expression in a strain of *Escherichia coli*. The successful expression of recombinant transglutaminase protein from Atlantic cod (AcTG‐1) as a soluble protein upon induction at low temperature was confirmed by sodium dodecyl sulfate/polyacrylamide gel electrophoresis, immunoblotting and mass spectrometry analysis. Biochemical characterisation demonstrated that the transglutaminase was active between 0 and 65 °C, but was completely inactivated after 20‐min incubation at 70 °C. Interestingly, the enzyme displayed cold‐adapted features, such as temperature instability combined with high catalytic efficiency at low temperatures (8–16 °C). In addition, the enzyme had optimal activity at 50 °C, a new feature for a cold‐adapted enzyme. AcTG‐1 was active in the pH range from 6 to 9, with an optimum at pH 8, and required 5 mm calcium for maximum activity. Potential calcium‐binding sites in the enzyme were predictable, making the enzyme an appropriate model for studying structure–function relationships in the calcium‐dependent transglutaminase family. *In vitro* gel analysis revealed that transglutaminase cross‐linked casein, collagen and gelatin. The binding of fish fillets in the presence of recombinant AcTG‐1 provided further macroscopic proof for the potential application of AcTG‐1 as a biological cross‐linker in the food industry. Once binding occurred, fish fillets withstood further processing such as frying, boiling, freeze‐thawing and chilling. The low‐temperature activity and new enzymatic properties of AcTG‐1 appear to offer advantages over commercially available enzymatic glues in the food industry.

## Graphical abstract

A gene encoding a transglutaminase from Atlantic cod has been used to successfully produce a recombinant protein. Biochemical characterisation demonstrates that it displays activity between 0 and 65 °C and is completely inactivated at after 20 min at 70 °C and calcium is needed to activate it. Binding of casein and fish fillet in the presence of recombinant transglutaminase shows that it acts as a biological cross‐linker.

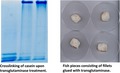

**Abbreviations**
Ac: Atlantic cod
Asp: aspartic acid
Asn: asparagine
Cys: cysteine
EDTA: ethylenediaminetetraacetic acid
FXIIIa: human blood coagulation factor XIIIa
His: histidine
HRP: horse radish peroxidase
IPTG: isopropyl β‐D‐1‐thiogalactopyranoside
K: lysine
MDC: monodansylcadaverine
Q: glutamine
Q‐TOF/LC‐MS/MS: quadrupole mass time‐of‐flight/liquid chromatography–tandem mass spectrometry
RT‐PCR: reverse transcription polymerase chain reaction
SDS/PAGE: sodium dodecyl sulfate/polyacrylamide gel electrophoresis
TG: transglutaminase

## Introduction

Transglutaminases (TGs; protein–glutamine y‐glutamyltransferases, http://www.chem.qmul.ac.uk/iubmb/enzyme/EC2/3/2/13.html) were first described in 1959 and found to form extensively cross‐linked, generally insoluble protein polymers 1, 2. These structures were important for organisms in the formation of skin barrier, hair growth, wound healing and blood clotting 3.

Transglutaminases are a family of enzymes that catalyse the cross‐linking of proteins by forming covalent bonds between glutamine (Q) and lysine (K) residues in different polypeptides. Such iso‐peptide bonds created by TG are highly resistant to proteolysis and increase the mechanical stability of the modified proteins 4. TGs have been found in a diverse range of organisms, such as bacteria, crustaceans, insects, fish, plants and mammals, which indicate that they play a key role in many biological processes 1, 5. Nine TGs have been described in humans and have been shown to be of clinical importance as they have been implicated in neurodegenerative diseases, tissue fibrosis, cancers and coeliac diseases 6, 7. All mammalian TGs, except a catalytically inactive homolog, require calcium for their catalytic activity and have the same amino acid sequence at their active site 4. Based on the structure and sequence of the enzymes’ active site, TGs belong to the superfamily of papain‐like cysteine proteases, all of which have the catalytic triad – cysteine (Cys), histidine (His) and aspartic acid (Asp), or cysteine (Cys), histidine (His) and asparagine acid (Asn) 4. Research has supported the idea that the deregulated expression and function of TG contributes to pathological conditions such as cancer metastasis, tissue fibrosis, coeliac disease and neurodegenerative disorders 8, 9. The coeliac disease is a gluten‐induced immune‐mediated disease characterised by a specific genetic genotype (HLA‐DQ2 and HLA‐DQ8 genes) and autoantibodies (anti‐tissue transglutaminase) 10. A key player is transglutaminase 2, which is able to modify gluten‐derived gliadin peptide 11. The deamination of the gliadin peptide results in increased binding affinity to the human leucocyte antigen (HLA) DQ2 and DQ8 molecules, triggering a strong immune response 11. In plants, TGs have been linked to the development of chloroplasts 12, while TGs in fish seem to be involved in immune defence 13.

The increasing interest in TGs also arises from their ability to cross‐bind proteins and to bind different proteins that are not normally linked. They can be used in most food processes containing proteins to prepare existing food products or to develop entirely new products and processing methodologies. Microbial transglutaminase is widely used in various food processes: to manufacture cheese and other dairy products, in meat processing, and to manufacture bakery products. The use of microbial transglutaminase in the processed food has raised some concerns, since microbial transglutaminase is a potential inducer of coeliac disease 14. Microbial transglutaminase imitates functionally to the endogenous tissue transglutaminase, the autoantigen of coeliac disease, and may therefore represent an inducer of coeliac disease 14. Although microbial TGs have been extensively used, fish‐derived TGs are especially interesting for their application in food processing. Organisms that live in cold environments mostly produce cold‐adapted enzymes 15. The high catalytic activity of these enzymes at low temperatures, coupled with their low thermal stability, make them attractive for applications where it is preferable to carry out the treatment under low‐temperature conditions, and when it is necessary to use a heat‐labile step in a multistep reaction to stop the reaction 15. Moreover, using cold‐adapted enzymes at low temperatures has the added benefits of low‐energy requirements and of protecting substrates and products from degradation. TG is used in the food industry; however, a commercial cold‐adapted counterpart is not presently available.

In fish, TGs have been studied in relation to egg maturation, optic nerve generation and immunity 13, 16, 17. However, despite investigations into the mechanisms of fish TGs, many of the fundamental biochemical and physiological properties remain elusive. An important step in the elucidation of the pivotal roles of TG has been the isolation of transglutaminase sequences from different organisms. In fish, two related complementary cDNA clones of TGs from Atlantic cod were isolated and named AcTG‐1 and AcTG‐2 13. We also found that these *AcTG* genes were upregulated by double‐stranded poly(I:C) synthetic double‐stranded RNA that simulates a viral infection, indicating that the enzyme plays an important role in antiviral defence in the Atlantic cod 13, 18.

In this study, AcTG‐1 was successfully expressed in *Escherichia coli* BL21 cells. We demonstrate that changing temperature conditions during expression was important for producing soluble active AcTG‐1. The enzyme was produced as a soluble protein with a concentration of 1 mg from 1 litre of expression culture and showed typical cold‐adapted features such as a higher catalytic efficiency at lower temperature combined with temperature instability. However, the temperature optimum differed from earlier published cold‐adapted features and showed peaks in activity at 8–16 °C and 50 °C. We further provide direct proof for potential uses of the cross‐linking enzyme in food production by binding casein, collagen, gelatin and fish fillets in the presence of recombinant AcTG‐1. This enzyme shows characteristics equivalent to eukaryotic TGs, in which calcium is essential for catalytic activity. Activity studies indicated calcium was essential for enzymatic activity and showed maximum activity at 5 mm. Using a consensus method (COACH), including sequence alignment, potentially important residues involved in calcium binding were identified. This work allows us to now study the relationship between the structure and activity of TGs and define uses for TG as a novel glue enzyme in the food industry.

## Materials and methods

### Construction of the expression plasmid for Atlantic cod TG‐1

Full‐length AcTG‐1 was cloned from the head kidney of Atlantic cod by a reverse transcription polymerase chain reaction (RT‐PCR) and rapid amplification of cDNA ends 13. The region encoding the *AcTG*‐1 gene was used as a template in a PCR to produce the plasmid, pET151/AcTG‐1. The AcTG‐1 cDNA sequence was amplified by PCR using the primer pairs: AcTG1‐F 5′‐CACCATGGCCCACACAAACCGTTTAA‐3′/AcTG1‐R 5′‐TCACAGATCCTCTTCTGAGATGAGTTTTTGTTCCCCGAATATGTTGGGCATCATG‐3′. The PCR product was excised from the gel and cloned into a pET151/D‐TOPO vector (Invitrogen, Carlsbad, CA). To confirm the PCR fragment contained the *TG*‐1 gene, sequencing with the T7 promoter/priming site, 5′‐TAATACGACTCACTATAGGG‐3′, and T7 reverse priming site, 5′TAGTTATTGCTCAGCGGTGG‐3′ (universal primers), was conducted. A polyhistidine tag was present in AcTG‐1 at the N terminus that allowed the detection of the protein using anti‐His antibody and purification with His‐Trap columns. A myc epitope at the C‐terminal end of AcTG‐1 was introduced during PCR.

### Small‐scale expression of His‐tag‐rAcTG‐1

The recombinant vector, pETAcTG‐1, was used to transform the bacterial expression strain *E. coli* BL21 (DE3) and was analysed for recombinant production using sodium dodecyl sulfate/polyacrylamide gel electrophoresis (SDS/PAGE), immunoblotting and mass spectrometry. Bacterial cells were induced at different temperatures after the addition of isopropyl β‐D‐1‐thiogalactopyranoside (IPTG) and harvested after 16‐h incubation. The cells were harvested by centrifugation at 3000 ***g*** for 20 min and frozen at –20 °C. Cells were then resuspended in lysis buffer (50 mm potassium phosphate, pH 7.8, 400 mm NaCl, 100 mm KCl, 10% glycerol, 0.5% Triton X‐100, 10 mm imidazole), lysed by freezing at –80 °C and thawing at 42 °C three times, incubated on ice with lysozyme (1 μg μL^−1^) for 1 h and sonicated. Cellular debris (pellet, insoluble fraction) was removed by centrifugation at 4 °C (4500 ***g***, 20 min) and a clear supernatant (soluble fraction) was achieved by filtration through a 45‐μm filter.

### Large‐scale expression and purification of His‐tag‐rAcTG‐1

Protein expression of His‐tag‐rAcTG‐1 was performed using *E. coli* BL21 (DE3) cells harbouring pET151/AcTG‐1 (rAcTG‐1) constructs grown in LB medium supplemented with 100 μg·mL^−1^ ampicillin at 37 °C to an OD600 of 0.5–0.8. Recombinant protein expression was induced with 1 mm IPTG at 13 °C for 16 h. The cells were harvested and lysed as described earlier. The filtered supernatant was applied to a 1 mL His‐Trap HP column (GE Healthcare, Little Chalfont, UK). The column was washed with wash buffer (25 mm HEPES, 300 mm NaCl, 10 mm imidazole, pH 7.5) before rAcTG‐1 was eluted using elution buffer (25 mm HEPES, 300 mm NaCl, 500 mm imidazole, pH 7.5). In all the following steps, fractions containing TG were determined by SDS/PAGE, mass spectrometry and immunoblotting. The His‐tag was cleaved off using an AcTEV Protease assay (Thermo Scientific, Waltham, MA) according to the manufacturer’s instructions and protein was concentrated using an ultrafiltration column (Amicon/Merck, Billerica, MA, USA).

### Electrophoresis and immunoblotting analysis

Protein samples were analysed by SDS/PAGE using a 12% polyacrylamide gel following the method of Laemmli 19. The gel was transferred at 15 mA for 1 h onto a nitrocellulose membrane using a Trans‐Blot apparatus. The membrane was thereafter incubated with a monoclonal antibody against the His‐tag (1 : 1000 dilution) as the primary antibody, and horse radish peroxidase (HRP)‐labelled anti‐mouse IgG diluted 1 : 5000 as the secondary antibody. Detection was performed using an enhanced chemiluminescence detection system.

### Mass spectrometry

Protein bands were excised by scalpel and analysed by the proteomic facility at the University of Tromsø, Norway. Protein samples were in‐gel‐digested using trypsin and proteins identified by quadrupole mass time‐of‐flight/liquid chromatography–tandem mass spectrometry (Q‐TOF/LC‐MS/MS). Data were searched against the EST vertebrate database using the Mascot engine.

### Protein concentration assay

The amount of protein was determined with a BCA protein assay kit (Thermo Scientific) using bovine serum albumin as a standard 20.

### Dependency of optimum AcTG‐1 activity on temperature, pH and calcium

Enzyme activities were assayed by a fluorometric method based on the incorporation of monodansylcadaverine (MDC) into N,N’‐dimethyl casein. The fluorescence intensity was assayed with the excitation wavelength setting at 350 nm and emission at 500 nm (Ex 350/Em 580) 21. The reaction was carried out in 1.2 mL of solution containing 15 μL MDC, 1 mg·mL^−1^ N,N′‐dimethyl casein, 5 mm CaCl_2_, 3 mm dithiothreitol and 50 mm Tris/HCl (pH 7.5) for 1 h. The reaction was terminated by the addition of 50 μL 0.5 m ethylenediaminetetraacetic acid (EDTA). The optimal temperature was assayed from 0 °C to 80 °C. TG activity was calculated according to Takagi et al. 22, 23. For assessing the optimal pH activity, buffers ranging from pH 3.0 to 10.0 were used (0.1 m sodium acetate/acetic acid for pH 3.0–6.0, 0.1 m imidazole/hydrochloric acid for pH 7.0–8.0 and 0.1 m carbonate/bicarbonate for pH 9.0–10.0). The thermostability of AcTG‐1 was determined by incubating the enzyme at various temperatures, ranging from 0 °C to 80 °C, for 20 min and measuring the residual activity of AcTG‐1 by fluorometry. The calcium dependency of the enzyme was tested by adding increasing amounts of Ca^2+^ (0–20 mm) and using the chelating agent, EDTA. All enzyme activity assays were performed in triplicate and the mean values were plotted against temperature and pH.

### Bioinformatics

A three‐dimensional model of AcTG‐1 was predicted using I‐TASSER (http://zhanglab.ccmb.med.umich.edu/I-TASSER/) 24. The COACH method, in addition to multiple alignment tools (ClustalX), was used to identify potential Ca^2+^‐binding residues.

### Cross‐linking of casein by AcTG‐1

The cross‐linking of casein by AcTG‐1 was detected by incubating 10 µL (0.1 µg µL^−1^) of enzyme extract and 10 µL of casein solution (8 mg·mL^−1^ casein, 5 mm CaCl_2_, 50 mm Tris/HCl pH 7.5, 300 mm NaCl) at 16 °C for up to 1 h and then separating the protein sample by SDS/PAGE. The effects of various reagents (5 mm CaCl_2_ and 5 mm EDTA) and the concentration of enzyme (0, 0.25 µg, 0.5 µg and 1.0 µg) on cross‐linking were tested.

### Cross‐linking of gelatin and collagen by AcTG‐1

The cross‐linking of fish gelatin and collagen by AcTG‐1 was detected by incubating 10 µL (0.1 µg µL^−1^) of enzyme extract and 10 µL of gelatin or collagen solution (8 mg·mL^−1^ gelatin or collagen, 5 mm CaCl_2_, 50 mm Tris/HCl pH 7.5, 300 mm NaCl) for up to 1 h and then separating protein samples by SDS/PAGE.

### Cross‐linking of fish fillets by AcTG‐1

Fresh fish fillets from cod were bought at the local food market. The experiments were performed in accordance with the Norwegian National Committees for Research Ethics. Four pieces of fish fillet (50 g) were incubated with AcTG‐1 (10 mL, 0.1 mg·mL^−1^), wrapped in plastic foil and incubated at 8 °C, overnight. The pieces of cod glued together into one piece were then sliced and subjected to various treatments such as refrigeration, freeze‐thawing, frying and boiling at 100 °C.

## Results

**Fig. 1:**
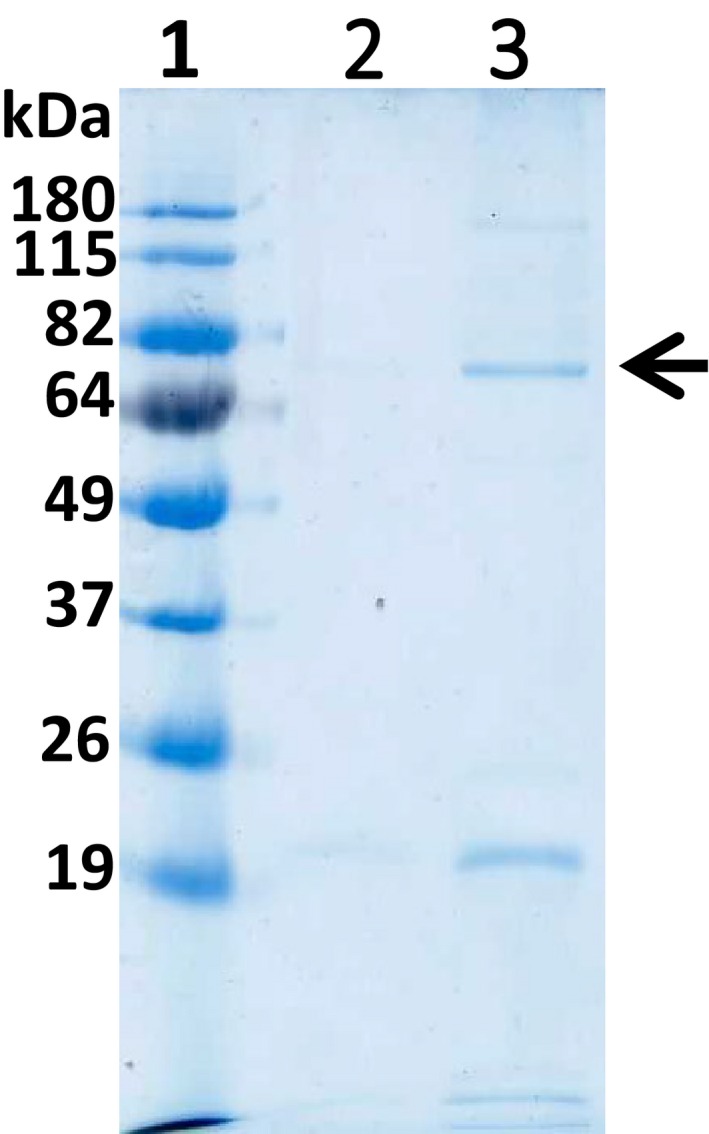
The SDS/PAGE analysis from the large‐scale production of AcTG1‐1 in *Escherichia coli* at 13 °C. Upon the large‐scale production of AcTG1‐1 in *E. coli* at 13 °C, fractions were collected from His‐tag chromatography and eluted with increasing concentrations of imidazole (150 mm; lane 3). Following the harvesting of protein extracts, the supernatant fraction was bound to the His‐tag column and washed with 10 mm imidazole (lane 2) before elution. The fractions were run on a 12% SDS/PAGE gel, 180 V for 1 h and stained with Coomassie Brilliant Blue. The numbers at the top indicate lanes and the molecular weights of the standards are indicated at the left margin. Lane 1: protein ladder (BenchMark Pre‐Stained); lane 2: wash fraction; and lane 3: elution fraction. The position of the AcTG‐1 is indicated by the arrow.

**Fig. 2:**
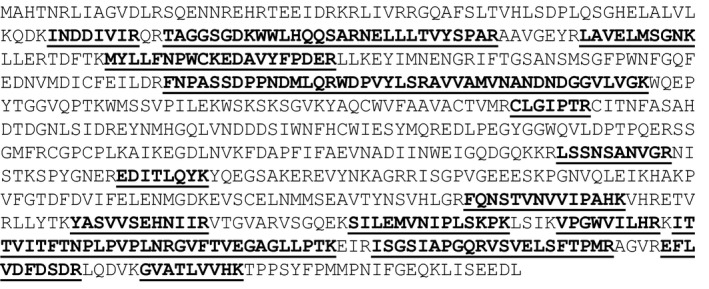
The MS data analysis of the purified AcTG‐1 enzyme. The amino acid sequence of AcTG‐1 is shown and detected fragments from MS analysis are shown in bold and underlined.

**Fig. 3:**
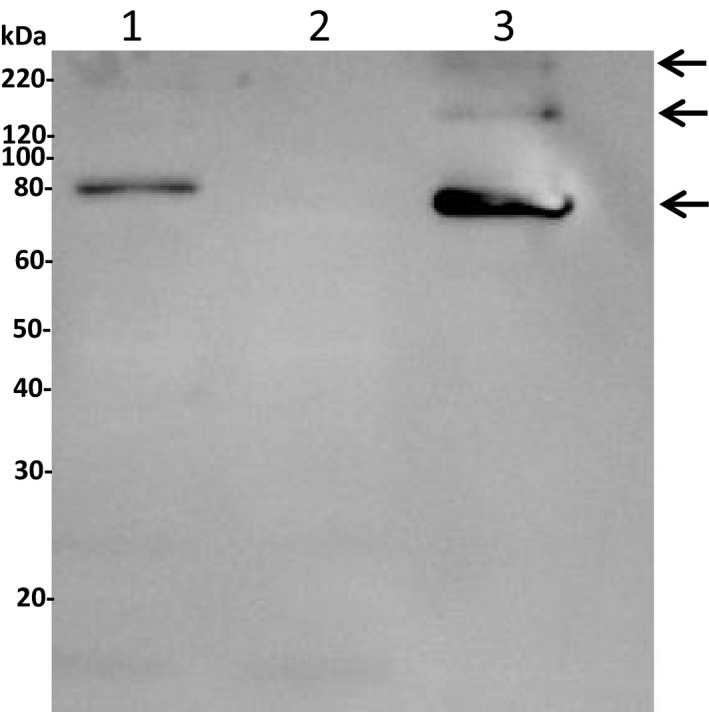
Gel blot analysis of AcTG‐1. For the immunological identification of AcTG‐1, an anti‐His antibody (1 : 3000) was used as a primary antibody and HRP‐conjugated goat anti‐mouse (1 : 5000) as a secondary antibody. The molecular weights of Magic Marker standards are indicated at the left margin. Lane 1: extract fraction; lane 2: elution fraction 1; and lane 3: elution fraction 3. The positions of positive AcTG‐1 bands are indicated by arrows.

**Fig. 4:**
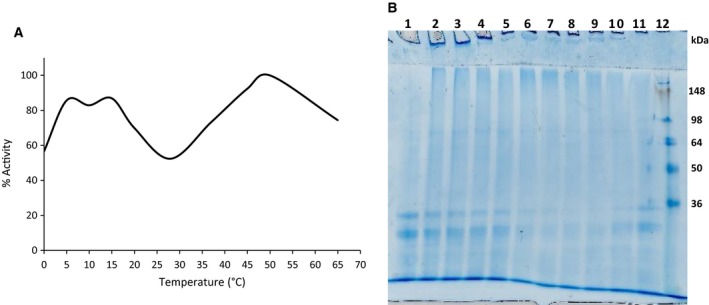
Influence of temperature on AcTG‐1 activity. The activity of AcTG‐1 was tested after 1 h of incubation at various temperatures and then stopped with the addition of EDTA (A). Data are given as the mean of three measurements and the highest value of the activity was set as 100%. Casein was separated by a 12% SDS/PAGE gel after incubation with TG (B). Samples of TG were incubated with casein for 1 h at 16 °C, and then, the reaction was stopped by the addition of EDTA. Samples used in a 12% SDS/PAGE gel analysis: lane 1: control (no TG); lane 2: 0 °C; lane 3: 5 °C; lane 4: 10 °C; lane 5: 15 °C; lane 6: 20 °C; lane 7: 28 °C; lane 8: 37 °C; lane 9: 45 °C; lane 10: 50 °C; lane 11: 65 °C; and lane 12: protein standard (SeeBlue Plus2 Pre‐Stained).

**Fig. 5:**
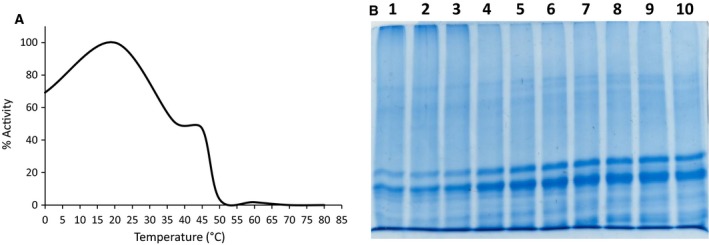
Temperature stability of AcTG‐1. AcTG‐1 was first incubated for 20 min at various temperatures and then for 1 h at 16 °C and its activity stopped by the addition of EDTA (A). Data are given as the mean of three measurements and the highest value was set as 100%. Casein was separated by a 12% SDS/PAGE gel after incubation with temperature‐exposed TG (B). Samples of TG were exposed to various temperatures for 20 min and then incubated with casein for 1 h at 16 °C and the reaction stopped by the addition of EDTA. Samples used in a 12% SDS/PAGE analysis. Lane 1: 0 °C; lane 2: 20 °C; lane 3: 37 °C; lane 4: 40 °C; lane 5: 45 °C; lane 6: 50 °C; lane 7: 60 °C; lane 8: 70 °C; lane 9: 80 °C; and lane 10: control (no TG).

**Fig. 6:**
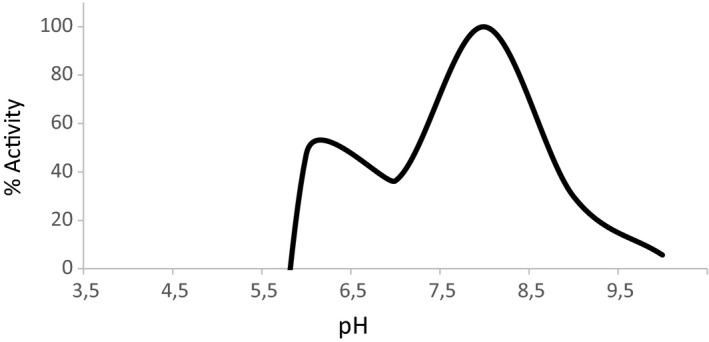
The pH dependency of AcTG‐1 activity. The activity of AcTG‐1 was tested after a 1‐h incubation at various pH levels and then stopped by the addition of EDTA. Data are given as a mean of three independent measurements, and the highest value was set as 100%.

Recombinant expression of the construct, pETAcTG‐1, in *E. coli* BL21 cells at 13 °C yielded a recombinant protein with a molecular weight of about 80 kDa upon protein purification and Coomassie Brilliant Blue staining after SDS/PAGE (Fig. 1, lane 3). The recombinant protein expressed in the soluble fraction was identified by 16 peptides using mass spectrometry (Fig. 2, peptides underlined). Immunoblot analyses showed recombinant AcTG‐1 as a band corresponding to the size of the predicted mature protein, indicating recombinant TG was not altered by proteolytic degradation (Fig. 3). Also, two higher molecular weight bands, with molecular weights of approximately 160 kDa and 240 kDa, were present. The temperature optimum for the enzymatic activity of AcTG‐1 was determined by measuring the fluorescence of MDC cross‐linking to N,N′‐dimethyl casein upon exposure of the purified enzyme to different temperatures (Methods). The Fluorolog activity assay showed that AcTG‐1 was active between 0 °C and 65 °C, with two maxima in the temperature range of 8–16 °C, and at 50 °C (Fig. 4). Catalytic activity was not detectable at a temperature of 70 °C or higher. In an instability analysis, AcTG‐1 was incubated at various temperatures for 20 min before testing for activity. This experiment showed that AcTG‐1 was stable up to a temperature of 20 °C. Above 20 °C, AcTG‐1 started to lose activity and was completely inactivated at 70 °C (Fig. 5). The pH dependency of AcTG‐1 was examined by incubation of the enzyme in various buffers titrated to the corresponding pH values. At a temperature of 16 °C, the purified AcTG‐1 showed activity between pH 6 and 10. At pH 8, AcTG‐1 reached its maximum activity; the activity decreased rapidly when the pH was below 6 and was completely lost at pH 5 and below (Fig. 6).

**Fig. 7:**
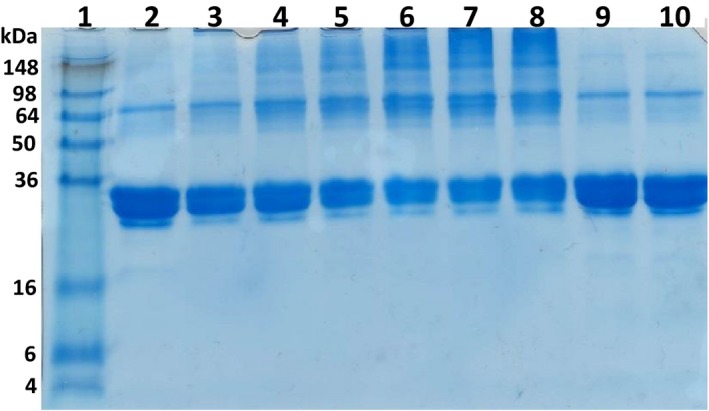
Cross‐linking of casein upon AcTG‐1 treatment. Casein was incubated for up to 60 min in the presence of AcTG‐1. Reactions were stopped by the addition of sample buffer and then analysed on a 12% SDS/PAGE gel. Separated proteins were visualised in the gel by Coomassie Brilliant Blue staining. Lane 1: protein ladder (SeeBlue Plus2 Pre‐Stained); lane 2: 0 min; lane 3: 2 min; lane 4: 5 min; lane 5: 10 min; lane 6: 20 min; lane 7: 40 min; lane 8: 60 min; lane 9: no calcium (60 min); and lane 10: calcium (5 mm) and EDTA (5 mm; 60 min).

**Fig. 8:**
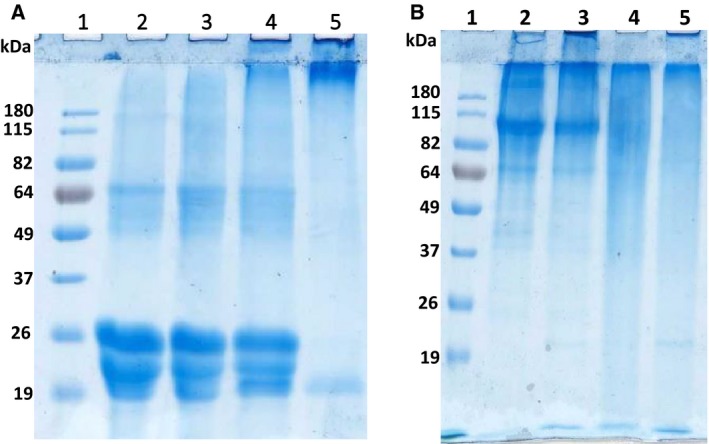
Cross‐linking of casein, collagen and gelatin upon AcTG‐1 treatment. Casein was incubated for 1 h with different concentrations of AcTG‐1 (A). Reactions were stopped by the addition of sample buffer and then analysed on a 12% SDS/PAGE gel. Separated proteins were visualised in the gel by Coomassie Brilliant Blue staining. Lane 1: protein standard (BenchMark Pre‐Stained); lane 2: no TG; lane 3: 0.35 μg AcTG‐1; lane 4: 0.5 μg AcTG‐1; and lane 5: 1 μg AcTG‐1. Fish collagen and gelatin were incubated for up to 60 min in the presence of AcTG‐1 (B). Reactions were stopped by the addition of sample buffer and then analysed on a 12% SDS/PAGE gel. Separated proteins are visualised in the gel by Coomassie Brilliant Blue staining. Lane 1: SeeBlue Plus2 marker; lane 2: collagen; lane 3: collagen with AcTG‐1; lane 4: gelatin; and lane 5: gelatin with AcTG‐1.

The cross‐linking activity of the enzyme was also studied by incubation of casein with AcTG‐1 for up to 1 h at 16 °C (Fig. 7). Electrophoresis of casein incubated with the enzyme extract showed that the intensity of casein decreased and the amount of cross‐linked casein products with higher molecular weight increased (Fig. 7, lanes 2–8). Three of the potential cross‐linked casein bands, with estimated sizes> 250 kDa, 125 kDa and 65 kDa, were analysed by MS. The proteins, α‐s1‐casein, α‐s2‐casein, β‐casein and κ‐casein, were identified in all bands, indicating that casein was cross‐linked by AcTG‐1. Moreover, the cross‐linking of casein protein bands was not observed in the absence of added calcium or in the presence of both calcium and EDTA (Fig. 7, lanes 9 and 10). The cross‐linking activity of the enzyme was further studied by the incubation of milk casein with different concentrations of AcTG‐1, and by incubating fish collagen or gelatin with AcTG‐1 for 1 h at 16 °C (Fig. 8). The electrophoresis of casein incubated with different concentrations of enzyme, from 0 up to 1 μg, clearly shows an increase in cross‐linked casein products and a decreased intensity of casein as a result of increasing enzyme concentration (Fig. 8 A). The electrophoresis of both collagen and gelatin from fish showed the same trend as casein, whereby incubation with enzyme resulted in cross‐linked products, but not at the same level as observed for casein (Fig. 8 B).

**Fig. 9:**
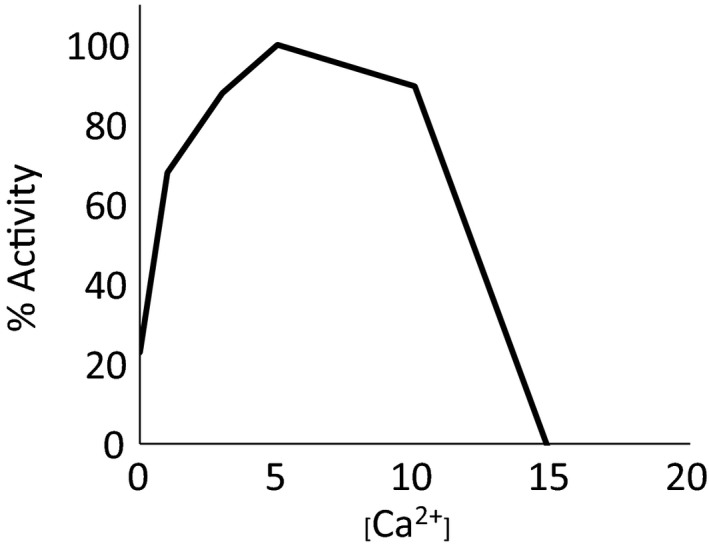
Examination of AcTG activity as a function of the concentration of calcium. The activity of TG was tested after a 1‐h incubation at various calcium levels and then stopped by the addition of EDTA. Concentrations of calcium used were 0, 1, 3, 5, 10, 15 and 20 mm. Data are given as a mean of three independent measurements and the highest value was set as 100%.

**Fig. 10:**
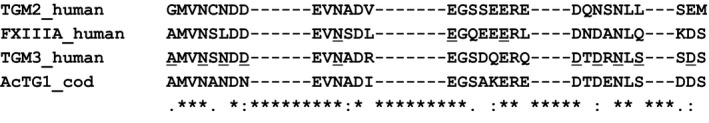
Multiple sequence alignments of calcium‐binding sites of transglutaminases. Sequence alignments of calcium‐binding sites of AcTG‐1 compared with other members of the transglutaminase family using ClustalW. The underlined bold characters indicate the amino acids that coordinate calcium binding in known crystal structures.

Further characterisation of AcTG‐1 activity showed that the enzyme was activated by calcium, with an optimum at 5 mm (Fig. 9). Without the addition of calcium, the activity was strongly compromised. Several potential calcium‐binding residues in AcTG‐1 have previously been identified (Asn‐392, Asp‐394, Glu‐441) 13. A comparison of mammalian TG sequences demonstrated that residues involved in calcium binding were conserved in AcTG‐1 (Fig. 10). Using a meta‐server approach to the prediction of protein–ligand binding sites, several new potential calcium‐binding residues were identified. These amino acids were Ala‐221, Asn‐224, Asn‐226, Asp‐228, Asp‐320, Asp‐322, Asn‐324, Ser‐326 and Glu 446 in addition to the earlier predicted Asn‐392, Asp‐394 and Glu‐441 (Fig. 10).

**Fig. 11:**
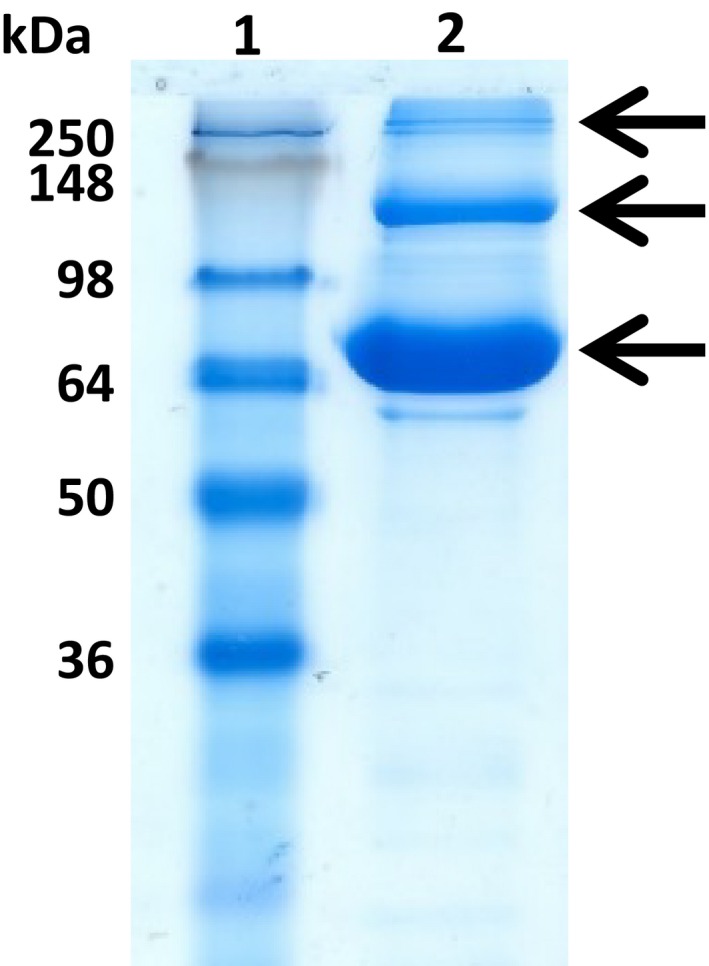
The SDS/PAGE analysis of concentrated AcTG1‐1. The fractions were run on a 12% SDS/PAGE gel at 180 V for 1 h and stained with Coomassie Brilliant Blue. The numbers at the top indicate lanes and the molecular weights of the standards are indicated at the left margin. Lane 1: protein ladder (SeeBlue Plus2 Pre‐Stained); and lane 2: concentrated AcTG‐1 (12 mg·mL^−1^). The positions of AcTG‐1 are indicated by the arrows.

Protein gel electrophoresis of concentrated, expressed AcTG‐1 with a cleaved His‐tag (12 mg·mL^−1^) also showed the existence of different forms that were all confirmed to be AcTG‐1 by MS analysis. Molecular weight predictions estimated the existence of monomeric, dimeric and trimeric versions of the AcTG‐1 (Fig. 11).

**Fig. 12:**
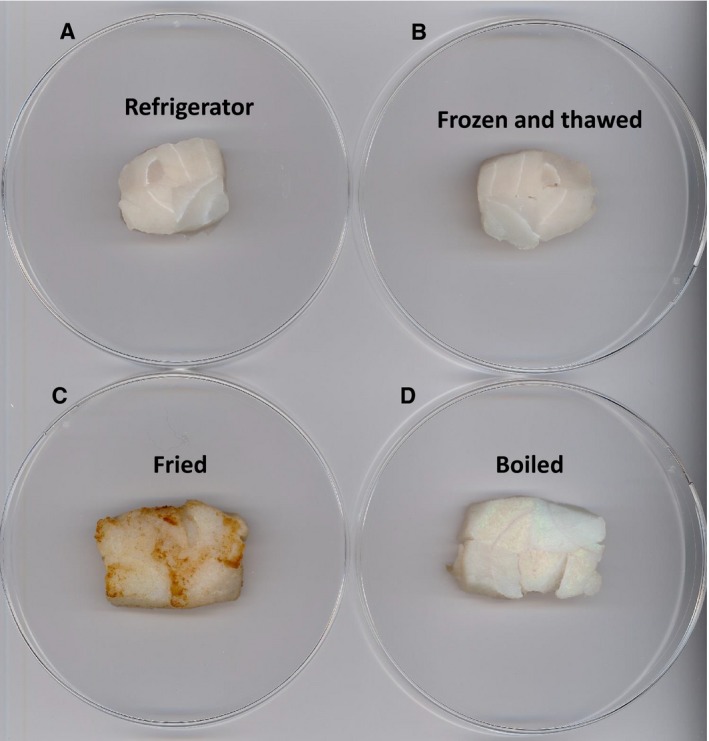
Cross‐linking of four different fish fillets subjected to AcTG‐1 treatment. After adding AcTG‐1 and incubating at 8°C, pieces of cod were glued together into one piece as shown above. The fish fillets were then subjected to different treatments: (A) refrigeration, (B) freeze‐thawing, (C) frying and (D) boiling at 100 °C.

Finally, AcTG‐1 was tested for its practical use in protein processing during food production. Four cod fillets were incubated with AcTG‐1. The fillets were wrapped in plastic foil and incubated overnight at a low temperature (8 °C). All the pieces of cod were found to be glued together into one piece that was then sliced and subjected to various treatments such as refrigeration, freeze‐thawing, frying and boiling at 100 °C. The experiments demonstrated that the enzyme effectively cross‐linked the fillets and that cross‐links between single pieces performed very well in terms of withstanding further processing, such as cooking, smoking, freezing or chilling, since binding was maintained in all the applied processes (Fig. 12).

## Discussion

We have succeeded in producing recombinant AcTG‐1 from Atlantic cod in *E. coli*. During growth of *E. coli* at 37 °C, the AcTG‐1 was found in the insoluble fraction and transglutaminase activity was not detected. However, when *E. coli* was grown at 13 °C, transglutaminase activity was clearly present. This finding demonstrated that the cloned AcTG‐1 cDNA encodes a catalytically active rAcTG‐1, although the active enzyme could only be harvested from transformants grown under restricted culture conditions. The full‐length sequence of AcTG‐1 cDNA, encoding a putative protein of 695 amino acids fused to its tag, had an estimated molecular mass of 80.3 kDa. The molecular weight of rAcTG‐1 was therefore similar to the predicted molecular weight of the mature form of AcTG‐1 protein. MS data and immunoblotting confirmed the identity of the band, while evidence of a breakdown of the recombinant protein by protease activity was not observed. However, two additional bands were observed in the immunoblot, with molecular weights indicating these may be higher molecular forms of the recombinant protein – probably dimer and trimer forms of AcTG‐1. Any potential oligomerisation was not, however, investigated further. A possible explanation for the increased yield of enzyme activity in the low‐temperature expression of recombinant AcTG‐1 is that there is a higher chance for correct folding of the enzyme, which may be based on a slower translation rate.

The transglutaminase activity of the purified enzyme was tested at different temperature and pH conditions and for temperature instability by measuring the incorporation of MDC into N,N`‐dimethylcasein. The enzymatic activity of prokaryotically expressed AcTG‐1 indicated that the enzyme did not absolutely require eukaryotic‐specific post‐translational modifications, a finding corresponding to that reported for red sea bream transglutaminase 23. The AcTG‐1 showed characteristic features of cold‐adapted enzymes: a high activity optimum at 8–16 °C, and substantial activity, even at 0 °C. However, an unexpected optimum was also observed with maximum activity at around 50 °C. This has never, to the authors’ knowledge, been described before. The peaks are in the same range that strongly indicates the same enzyme. An explanation for the three optima may be the existence of monomeric, dimeric and trimeric versions of AcTG‐1, each with its own optimum. Consequently, the model proposed here gives a new view on how oligomeric status affects both the stability and activity of enzymes. An oligomer version (dimer and trimer) may affect the enzyme stability compared with monomeric version 25. Monomeric version seems to be less stable and thermodynamically labile at high temperature compared with oligomer associations of protein/enzyme subunits 26. Furthermore, oligomer association of enzymes can also provide enzyme with changed temperature dependent activity due to the multivalent binding and increased stability 25. Cold‐adapted enzymes are characterised by high specific activity at low temperature and temperature lability at high temperature. These features of cold‐adapted enzymes are a trade‐off between stability and flexibility of the enzyme, where flexibility is necessary for optimal activity at low temperature. The optimum at 50 degrees for transglutaminase from Atlantic cod is unexpected since the organism lives mostly in the cold environment conditions. The reason for the optimum 50 degrees for transglutaminase activity in evolutionary terms could be explained by the stability of the enzyme is still intact and not lost completely in trade for flexibility.

Stability measurements showed a loss of activity above 20 °C and complete inactivation upon a 20‐min incubation at 70 °C, indicating that the protein was heat‐labile. The AcTG‐1 enzyme catalysed a cross‐linking reaction with an optimum at a cold temperature that is inactivated at even moderate temperatures. This leads us to the conclusion that the enzyme may be highly suitable for the food industry where processing at low temperatures is necessary. The use of cold‐adapted AcTG‐1 in the industry may also be advantageous because its highly specific activity at low temperatures reduces the amount of enzyme needed and because of its simple method of inactivation. Using enzymes active at low temperatures also provides benefits such as low‐energy requirements and the protection of substrates and products from degradation. Organisms that live in cold environments have evolved to express proteins capable of coping with reduced reaction rates at lower temperatures 27. Enzymes from such organisms are often characterised by high catalytic activity at low temperatures coupled with low thermal stability 15. The molecular mechanisms for cold‐adapted enzymes are not fully understood, but they are clearly linked to a protein structure that allows greater flexibility for catalysing enzymatic reactions at cold temperatures 28. Cold‐adapted enzymes tend to require a lower number of weak bonds, such as hydrogen bonds, and fewer specific interactions between regions, compared with proteins from organisms that grow best at higher temperatures 29. These features may explain the proteins’ flexibility in the cold and their inactivation at even moderate temperatures. The increased ocean water temperature could affect the activity of the fish transglutaminase in Atlantic cod *in vivo*, where a major concern would be the stability of the fish transglutaminase. *In vitro* characterisation showed that the Atlantic cod transglutaminase displays activity between 0 and 65 °C and is stable up to a temperature of 20 °C. Above 20 °C, it started to lose activity and was completely inactivated at 70 °C.

The optimal pH of AcTG‐1 was determined at different pH values. The optimum for AcTG‐1 activity was found to be at pH 8. However, AcTG‐1 also showed some activity at pH 6 and 9 so can be considered to be stable in this pH range.

Interestingly, Atlantic cod TG‐1 also displayed characteristics equivalent to mammalian TGs, with a calcium dependency shown for catalytic activity. The calcium‐bound crystal structures of mammalian TG are known from studies on the human blood coagulation Factor XIIIa (FXIIIa) and the epidermal transglutaminase, TG3 30, 31. FXIIIa has one Ca^2+^‐binding pocket while TG3 has three Ca^2+^‐binding sites 30, 31. The crystal structure of transglutaminase 2 (TG2) is also known, but the protein was crystallised in the absence of Ca^2+^ ions. The need for Ca^2+^ ions for TG2 reactions has been shown by *in vitro* analysis 25. However, the number of ions, their location in the TG2 structure and the role they play in activity and stability are still not understood 32. With respect to medical applications, the vast majority of research has been done on calcium‐dependent TG2, found in the tissues of animals and humans 33. The TG2 protein has been implicated in many serious diseases, such as cancer, neurodegenerative diseases, tissue fibrosis and coeliac diseases 34, 35, 36. Of interest is the fact that the TG2 gene is also transiently activated by poly(I:C) treatment, similar to our finding in Atlantic cod 13, 37. Our results confirm the calcium dependency of AcTG‐1 for activation of the enzyme. The optimum calcium concentration, within a range of 0–20 mM, was determined to be 5 mM. Higher concentrations of calcium seem to affect substrate molecules, probably causing precipitation of these molecules and making them unavailable for enzymatic reactions.

AcTG‐1 treated casein yielded bands at the top of the gel that were heterogeneous and tended to broaden. It is anticipated that casein polymerisation via intermolecular cross‐linking may alter the hydrophobic/hydrophilic balance of casein and change its solubility in the separation medium, leading to various aggregation states with different protein mobilities. Bands of cross‐linked casein were not detected in the absence of added calcium or in the presence of both calcium and EDTA. The cross‐linked casein bands resulting from AcTG‐1 cross‐linking were not affected by SDS or dithiothreitol. Under the denatured and reduced conditions of SDS/PAGE, covalently linked (Q‐K) casein was detectable. The casein from bovine milk is a phosphoprotein which consists of four main types: α‐s1, α‐s2, β and κ. Cross‐links were made by all four main types and the incorporation of AcTG‐1 itself was not detected by MS data. Furthermore, both fish collagen and gelatin seem to be suitable substrates for AcTG‐1, but were not as good as milk casein. The calcium dependency of TG may restrict the use of this enzyme where the calcium concentration is low or in the presence of Ca^2+^‐binding compounds such as EDTA. Many food proteins precipitate in the presence of calcium and the need for calcium in applications of TG in food processing on an industrial scale could therefore be challenging 38. By comparing mammalian TG sequences and using a meta‐server approach to the prediction of protein–ligand binding sites, several potential Ca^2+^‐binding residues were identified, indicating again that TG is a Ca^2+^‐binding protein that requires Ca^2+^ for activity. The amino acids identified were Ala‐221, Asn‐224, Asn‐226, Asp‐228, Asp‐320, Asp‐322, Asn‐324 Ser‐326, Asn‐392, Asp‐394, Glu‐441 and Glu‐446. These twelve residues appear to be good candidates for Ca^2+^‐binding sites in the molecule. A detailed understanding of TG–calcium binding will provide insights into this very specific aspect of protein–calcium interaction. It will also permit the rational design of new enzymes and the engineering of TG for applications in both food processing and medicine. In the case of overexpressed AcTG‐1, cod fillets were effectively glued together and subjected to various treatments, such as refrigeration, freeze‐thawing, frying and boiling at 100 °C. Once the binding occurred, the food product was stable during further processing, such as cooking, freeze‐thawing, chilling and frying. Strong, irreversible binding seems to be maintained in all these processes confirming proof of concept for the use of AcTG‐1 as a novel food glue enzyme.

In conclusion, AcTG‐1 shows transglutaminase activity and has characteristic cold‐adapted features. This trait may be beneficial for terminating the enzyme’s reaction by heat inactivation in biotechnology applications. Our biochemical analysis provides insights into the use of this enzyme in industrial applications for cold‐adapted transglutaminase enzymes. Our findings will also add to the scientific understanding of TG and contribute to understanding the role of calcium in regulating TG activity. This knowledge may shed light on the development of TG inhibitors that can, for instance, be used in the treatment of human diseases characterised by TG overexpression, such as coeliac disease, Huntington’s disease, Alzheimer’s disease and fibrosis 39, 40. At present, we are continuing our investigations into this fascinating enzyme and its potential use in the food industry and medicine.

## Conflict of interest

The authors declare no conflict of interest.

## Author contributions

CF conceived and designed the project; RGA, PK, IEL, RJB and CF acquired the data; RGA, PK, IEL, RJB, LAE and CF analysed and interpreted the data; and RGA, PK, LAE and CF wrote the paper.
